# Climate-driven restructuring of sediment microbiomes and ecosystem functions in aquaculture systems

**DOI:** 10.3934/microbiol.2026006

**Published:** 2026-03-30

**Authors:** Antonia Mataragka, Einar Ringø, Anastasios Klavdianos Papastathis

**Affiliations:** 1 Laboratory of Anatomy and Physiology of Farm Animals, Department of Animal Science, School of Animal Biosciences, Agricultural University of Athens, 75 Iera Odos Str., 11855 Athens, Greece; 2 Norwegian College of Fishery Science, Faculty of Biosciences, Fisheries and Economics, UiT, The Arctic University of Norway, Tromsø, Norway; 3 Institute for Fundamental Biomedical Research, Biomedical Sciences Research Center “Alexander Fleming”, Fleming 34, 16672 Vari, Greece

**Keywords:** aquaculture, climate change, sediment microbiome, antibiotic resistance, ecosystem resilience

## Abstract

Aquaculture expansion is occurring under accelerating climatic pressure. Warming, marine heatwaves, deoxygenation, salinity fluctuation, and intensified nutrient loading act simultaneously in aquaculture sediments, altering redox gradients and substrate fluxes that structure microbial communities. These stressors strengthen deterministic environmental filtering, reorganize interaction networks toward reduced-state dominance, and redistribute functional investment within sediment microbiomes; the biogeochemical engines regulating nutrient cycling, water quality, and disease dynamics. Such restructuring is associated with altered nitrogen processing, modified greenhouse gas fluxes, sulfide accumulation, enhanced pathogen performance, and enrichment of antimicrobial resistance determinants, with direct implications for production stability and disease risk. Evidence is synthesized to integrate quantified environmental forcing, ecological assembly mechanisms, and molecular functional responses into a unified framework linking microbial restructuring to ecosystem performance and operational resilience. Structural and functional microbial indicators suitable for early detection of redox compression and functional destabilization are evaluated, alongside resilience-oriented strategies spanning ecological design, microbiome management, engineering control, and adaptive monitoring. Despite substantial empirical progress, major gaps remain in resolving compound-stressor interactions, temporal reversibility, cross-system threshold comparability, and predictive modeling of microbial assembly under multi-driver forcing. Addressing these gaps is essential for developing mechanistically grounded, climate-resilient aquaculture systems.

## 1. Introduction

Aquaculture is one of the fastest-growing food production sectors globally and now supplies more than half the aquatic animal protein consumed worldwide [1]. Continued expansion is occurring under accelerating climatic pressure. Rising temperatures and increasing marine heatwave frequency [2],[3], progressive ocean deoxygenation [4], salinity restructuring across coastal and estuarine systems [5], and nutrient intensification associated with production systems [6],[7] no longer act as isolated disturbances. In aquaculture environments, these drivers co-occur and interact, reshaping sediment physicochemical conditions through altered oxygen availability, ionic gradients, and organic matter deposition.

Sediment microbiomes function as central biogeochemical processors within aquaculture systems whose climate sensitivity has been emphasized across terrestrial and aquatic systems [8], and they regulate organic matter mineralization, nitrogen transformations, sulfur cycling, and greenhouse gas exchange [9]–[13]. The sediment microbiomes also serve as environmental reservoirs of opportunistic pathogens, including *Vibrio* spp., *Aeromonas* spp., and *Pseudomonas* spp., as well as antimicrobial resistance determinants [14]–[16]. These functions are structured along steep redox gradients governed by oxygen penetration depth, electron acceptor distribution, and substrate flux [17],[18]. Climatic and production-related pressures directly modify these governing variables.

Experimental and observational studies have largely examined individual stressors in isolation and revealed that: Warming alters metabolic rates and oxygen solubility [3]; deoxygenation constrains aerobic processes [4]; salinity gradients impose compositional partitioning [5],[19]; and organic enrichment intensifies anaerobic pathways [20],[21]. Moreover, aquaculture sediments rarely experience single-driver exposure. Instead, thermal, redox, nutrient, and hydrodynamic forces operate simultaneously, producing compound environmental regimes (compound stressors) whose combined effects are not sufficiently integrated across studies.

Critical limitations, however, remain in how environmental drivers are quantified, integrated, and translated into functional and operational inference. First, stressor quantification is often inconsistent across systems, limiting cross-context comparison of effect magnitude and threshold behavior. Temperature increases are typically reported precisely, whereas oxygen minima duration, salinity fluctuation amplitude, and nutrient loading intensity are less standardized. Second, microbial responses are frequently described at the compositional level without systematic integration of ecological assembly dynamics, interaction restructuring, and functional redistribution into a coherent framework linking environmental forcing to system-level outcomes. Third, microbial indicators are increasingly proposed for monitoring, yet they are rarely embedded within a structured architecture that connects quantified stressors, ecological restructuring, measurable biogeochemical shifts, and operational resilience.

In this review, we address how climate resilience in aquaculture depends on maintaining sediment microbial functional buffering capacity under interacting environmental pressures. Evidence across warming, hypoxia, salinity restructuring, and nutrient intensification are synthesized; (i) document quantified environmental forcing and immediate microbial responses, (ii) examine the ecological and molecular mechanisms underlying community restructuring, (iii) evaluate measurable consequences for ecosystem functioning and production stability, (iv) identify structural and functional microbial indicators suitable for early detection of instability, and (v) assess system-level strategies for buffering microbial and biogeochemical performance.

By integrating stressor quantification, ecological mechanism, measurable functional output, and management translation across marine, brackish, and freshwater aquaculture systems, we aim to provide a mechanistically grounded and operationally relevant framework for understanding sediment microbiome stability under accelerating climatic change.

## 2. Realistic climate pressures and their direct effects on sediment microbes

### 2.1. Warming and marine heatwaves

Anthropogenic warming, the increase in Earth's average surface temperature caused directly or indirectly by human activities primarily through greenhouse gas emissions (CO_2_ and CH_4_), significantly increase marine heatwave probability and intensity. Severe events are approximately three times more likely at +1.5 °C global warming and nearly twenty-five times more likely at +3.5 °C [3]. Moreover, long-term records indicate that a ~1.5 °C increase in North Atlantic sea surface temperature over 54 years correlates with increased abundance of pathogenic *Vibrio* species, particularly *V. vulnificus*, *V. parahaemolyticus* and *V. alginolyticus*, which include opportunistic pathogens of fish, shellfish, and humans [15].

At sediment scale, a long-term heated coastal basin exposed to artificial thermal discharge for approximately 50 years exhibited bottom-water temperatures ~6 °C higher than those in a nearby unheated reference basin with comparable environmental characteristics used as the control [22]. In incubations spanning 6–35 °C, dissolved oxygen declined significantly with increasing temperature (ANOVA F₉,₃₈ = 218.3, P < 0.001). Bacterial production peaked at 28 °C and showed a significant bay × temperature interaction (F₁,₃₈ = 9.6, P = 0.037), with divergence between sediments originating from the long-term heated basin and those from the nearby unheated reference basin used as the control across the 16–35 °C incubation range (day 9; F₁,₃₈ = 19.54, P < 0.0001). Shannon diversity declined significantly across the temperature gradient (F₉,₅₆ = 27.06, P < 0.001), and community variation was associated with oxygen, NH_4_^+^, and Fe^2+^ concentrations (PERMANOVA P ≤ 0.041) [22]. Seasonal coastal temperature ranges of 3–22 °C have also been reported [23].

Short-duration laboratory microcosm incubations of pond sediments using discrete temperature treatments (4, 10, 15, 25, 30, and 35 °C) showed significantly elevated CO_2_ emission rates at 35 °C compared with lower temperature treatments [12].

Temperature-associated restructuring in aquaculture ponds coincided with shifts in ARG composition, including detection of *aadA*, *blaTEM*, *catB*/*catB3*, *dfrA1*, *ermB*, *mecA*, *qnrA*, *sul1*/*sul2*/*sul3*, *tetM*, *tetO*, *tetT*, and *tetW*. Resistome variation was associated with temperature, dissolved oxygen, and nitrate [24].

Across systems, warming is therefore associated with quantifiable oxygen decline, reduced alpha diversity, altered production dynamics, temperature-linked beta-diversity shifts, elevated CO_2_ emission at upper thermal treatments, and restructuring of antibiotic resistance gene (ARG) assemblages.

### 2.2. Hypoxia and deoxygenation

Ocean deoxygenation is progressing globally [4], and aquaculture ponds frequently operate under reduced oxygen regimes. Experimental pond exposures have defined normoxia (~17 kPa), constant hypoxia (~8 kPa), and diel oxygen minima approaching 0 kPa [25]. In coastal sediment systems, hypoxia is commonly defined as dissolved oxygen below 2 mg·L^−1^ [26].

Manipulation of oxic versus oxic–anoxic treatments generated significant dissolved oxygen contrasts (7.89 ± 0.06 mg·L^−1^ vs 7.49 ± 0.11 mg·L^−1^; p = 0.035) and concurrent temperature differences (29.13 ± 0.12 °C vs 29.81 ± 0.01 °C; p = 0.004) [27]. Oxygen treatment explained 37.6% of community variation, and Shannon diversity was significantly higher under oxic conditions [27].

Field comparisons between shallow and deep coastal sediments under oxygen deficiency showed larger compositional restructuring at the deeper hypoxic site, with relative abundance shifts up to 11% compared to 1.1% at the shallow site. This coincided with a 36% increase in organic matter content [21]. Sulfate-reducing bacteria, particularly members of the Desulfobacterota lineage (formerly Deltaproteobacteria) such as *Desulfobacteraceae* and related sulfate-reducing taxa, increased in relative abundance under oxygen limitation [21]. Seasonal oxygen and nutrient oscillations explained approximately 17–20% of total community variance (PERMANOVA R^2^ ≈ 0.17–0.20, P = 0.001) [28].

Under reduced oxygen conditions in fish-farm sediments, acetate concentrations reached up to 4.7 mM during summer and accounted for >99% of measured short-chain fatty acids [6]. In clam aquaculture sediments, nitrate reduction pathways showed differential routing between denitrification and dissimilatory nitrate reduction to ammonium (DNRA) under oxygen-limited conditions [18].

Oxygen limitation is thus associated with statistically supported community restructuring, enrichment of sulfate reducers, variance explained by oxygen–nutrient oscillations, accumulation of fermentation intermediates, and measurable shifts in nitrate reduction routing.

### 2.3. Salinity intrusion and fluctuation

Salinity gradients across freshwater, brackish, and marine sediments are consistently associated with significant microbial community differentiation.

Across 35 sediment systems (>2.3 million sequences), salinity explained a substantial fraction of community separation variance, and alpha diversity decreased from freshwater to marine sediments [5]. Metagenomic surveys confirmed statistically significant community differentiation along conductivity gradients [29]–[31].

Marine–brackish comparisons demonstrated significant beta-diversity separation along salinity gradients [19],[32]. In long-term basin monitoring, salinity was significantly associated with community variation alongside temperature and oxygen (PERMANOVA P ≤ 0.041) [22].

Marine sediments showed enrichment of sulfate-reducing Desulfobacterota, whereas freshwater sediments showed greater representation of nitrifier-associated taxa [5].

In aquaculture-linked marsh-to-pond conversion systems, methanogen co-occurrence simplification was statistically significant [13]. Concurrent declines in soil organic carbon (~17.6 to 6.97 g·kg^−1^) and the carbon-to-nitrogen (C/N) ratio (~10.85 to 5.66) were reported under brackish aquaculture conversion [33].

ARG assemblage differentiation across environmental gradients that include salinity has also been documented in aquaculture ponds [24],[34].

Explicit Practical Salinity Unit (PSU) increment magnitudes are inconsistently reported across studies, limiting direct cross-system effect-size comparison [5],[13],[22].

### 2.4. Compound stressors

In aquaculture sediments, warming, oxygen limitation, organic enrichment, nutrient loading, and salinity shifts frequently co-occur. Multivariate analyses indicate that community structure and resistome composition respond to interacting thermal, redox, and nutrient gradients rather than isolated drivers [22],[24],[28],[34].

Enriched hypoxic sites consistently exhibit larger compositional shifts and increased representation of anaerobic functional groups than non-enriched comparators [21].

Across systems, climate-relevant forcing narrows vertical redox stratification and strengthens anaerobic guild representation. For clarity, we refer to the progressive contraction of oxic layers and dominance of reduced-state processes under interacting stressors as redox compression. In the subsequent sections, we examine the ecological mechanisms underlying these shifts and their measurable functional consequences.

## 3. Mechanisms of microbial community change

### 3.1. Shifts in community assembly processes

The environmental gradients documented in Section 2 translate into measurable changes in ecological assembly dynamics. Community structuring can be partitioned into deterministic selection, stochastic drift, dispersal limitation, and historical contingency [35],[36].

Oxygen manipulation experiments demonstrated that treatment explained 37.6% of community variation, and Shannon diversity was significantly higher under oxic conditions [27], indicating oxygen availability as a dominant deterministic filter. Seasonal oxygen–nutrient oscillations explaining 17–20% of variance [28] further support strong environmental selection along redox gradients.

Short-term warming strengthened deterministic clustering and homogeneous selection, with network destabilization (R^2^ = 0.87, P < 0.01) and βNTI values exceeding +2 under thermal stress [12]. βNTI > +2 is consistent with dominance of variable selection rather than stochastic turnover.

Salinity transitions similarly impose deterministic species sorting across ionic tolerance ranges [5]. Across freshwater–marine gradients, compositional separation follows predictable environmental partitioning rather than neutral drift.

Dispersal limitation operates within vertically stratified sediments where steep redox gradients restrict microbial exchange between oxic and anoxic microzones. In aquaculture ponds, organic loading and hydrodynamic modification further constrain dispersal connectivity, reinforcing localized deterministic filtering.

Long-term heated sediments exhibit transcriptomic convergence during short-term warming exposure [22], suggesting stabilization of alternative functional baselines shaped by chronic exposure history. This demonstrates trajectory-dependent assembly under sustained forcing and highlights the influence of historical contingency.

Collectively, evidence across warming, oxygen limitation, and salinity restructuring indicates a shift from mixed stochastic–deterministic regimes toward stronger deterministic environmental selection under intensified stress.

### 3.2. Disruption of microbial interaction networks

Environmental restructuring extends beyond taxonomic turnover to modification of interaction topology.

Redox shifts reorganize carbon and electron flow across sediment consortia. In subarctic marine sediments, ^13^C-labeled substrates were incorporated into sulfate-reducing *Deltaproteobacteria* within 10 days [37], demonstrating rapid routing of labile carbon toward terminal anaerobic guilds. Metatranscriptomic analyses show substantial allocation of transcripts to sulfur energy metabolism [17], reflecting strong energetic investment in anaerobic metabolic pathways. Oxygenation events triggered rapid succession from genus *Desulfatiglans* to *Arcobacter* [26], illustrating reorganization of dominant functional guilds under changing redox conditions.

Under oxygen limitation and enrichment, interaction networks shift toward anaerobic consortia dominated by fermenters, sulfate reducers, and methanogens. Simplification of methanogen co-occurrence structures under brackish pond conversion [13] indicates reduced modular complexity under altered salinity and redox regimes.

Co-occurrence analyses demonstrate simplification of interaction topology and redistribution of network centrality under stress [38],[39]. Nutrient enrichment modifies network centrality and interaction strength among dominant taxa [40],[41], indicating redistribution of interaction hubs under loading pressure.

Aquaculture-associated microbiome surveys show that enrichment regimes shift dominant taxa while reducing network connectivity [42],[43]. Thermal stress produces parallel increases in clustering and reduced connectivity [12], reinforcing consolidation around stress-tolerant consortia.

### 3.3. Genetic and functional adaptations

At molecular resolution, environmental forcing is expressed through redistribution of metabolic pathways, gene abundance, and functional allocation [8].

Under redox compression (Section 2.4), anaerobic respiration and sulfur cycling are favored. Sulfate reduction is mediated by *dsrAB* and *aprAB*, whereas sulfur oxidation involves *sox* gene clusters [10]. Expanded diversity of sulfur-cycling genes across marine sediments supports enhanced functional investment in sulfur metabolism under oxygen-limited conditions. In active sediment metatranscriptomes, approximately 7% of bacterial transcripts are allocated to sulfur energy pathways [17], indicating substantial energetic prioritization of reduced-state processes. Warming further amplifies these dynamics by increasing metabolic rates while reducing oxygen solubility [3],[12], thereby compressing oxic–anoxic zonation and promoting sulfate-reducing dominance in surface sediments [6],[9].

Nitrogen transformation pathways exhibit redox-sensitive redistribution. Oxygen limitation shifts nitrate reduction routing between denitrification (*nirK*/*nirS*, *nosZ*) and DNRA (*nrfA*) [18]. Metagenomic surveys confirm differential representation of nitrogen cycling genes across environmental gradients [44]–[48], demonstrating functional reallocation under warming and enrichment.

Temperature-driven trait filtering further modifies competitive hierarchies and metabolic performance. Shifts in thermal optima (Tmin) influence carbon allocation patterns and pathway dominance under warming regimes [49], reinforcing selective stabilization of stress-tolerant metabolic modules.

Multi-omics approaches provide quantitative resolution of these adaptations. Marine sediment metaproteomics identified 823 protein groups and 6,179 unique peptides, with approximately 9% assigned to energy production and conversion [50]. Untargeted metabolomics demonstrated multivariate separation under nutrient enrichment, and δ¹⁵N tracing confirmed redistribution of aquaculture-derived nitrogen across trophic compartments [51]. Targeted metabolomics quantified 146 metabolites exhibiting concentration–response modulation under defined gradients [52], supporting pathway-level sensitivity to environmental forcing.

ARG dynamics represent an additional dimension of functional restructuring. Environmental gradients promote co-selection between stress tolerance traits and resistance determinants [24],[34]. Nutrient–metal interactions are associated with enrichment of multiple ARG classes [14]. ARGs frequently co-occur with mobile genetic elements, including integrons, transposases, and plasmid-associated sequences [14],[16],[53]–[55], reflecting broader patterns of bacterial genome plasticity and horizontal gene exchange [56]. The correlation between class 1 integrons (*intI1*) and total ARG abundance across impacted sediments supports mobilome-associated organization of resistance reservoirs.

These stressors therefore act not only on community composition but also on pathway allocation, redox-sensitive gene redistribution, metabolic trait filtering, and mobilome-linked resistance expansion.

### 3.4. The role of priority effects and legacy impacts

Historical contingency modifies present-day microbial responses to environmental forcing.

Sediments from a coastal basin chronically warmed for ~50 years by thermal discharge exhibited distinct transcriptomic baselines compared with sediments from a nearby unheated reference basin. Partial convergence during short-term warming exposure indicates persistent functional reconfiguration associated with long-term thermal history [22].

Organic enrichment reinforces legacy effects by maintaining reduced sedimentary conditions beyond the duration of external forcing. Rapid routing of labile carbon to sulfate-reducing guilds [37], combined with sustained allocation to sulfur metabolism [17], supports stabilization of anaerobic metabolic configurations across disturbance cycles.

Priority effects arise when early colonizers under altered oxygen or salinity regimes pre-empt niche space and influence later successional trajectories. In marsh-to-pond conversion systems, early restructuring of methanogen assemblages [13] may constrain subsequent community development.

Repeated stress can embed resistance determinants within stabilized community configurations [14]. Longitudinal datasets demonstrate disturbance-history imprinting at both taxonomic and functional levels [21],[32],[40],[41], and cross-system comparisons show persistence of gene-level differences beyond immediate disturbance events [48],[57]. In aquaculture sediments, priority effects and legacy impacts may influence post-disturbance recovery trajectories across production cycles [21],[32],[40]. Early colonizing taxa following fallowing or organic loading events can establish metabolic dominance that constrains subsequent community reassembly. Historical exposure to hypoxia or enrichment may therefore determine whether sediments return to pre-disturbance functional states or stabilize in reduced-capacity configurations [22],[41]. Such trajectory dependence directly influences system resilience under repeated climatic stress.

Environmental forcing therefore shapes not only immediate assembly but long-term trajectory stabilization through legacy effects, priority dynamics, and molecular imprinting.

## 4. Consequences for aquaculture ecosystem functioning

### 4.1. Disruption of biogeochemical cycling

Climate-driven restructuring of sediment microbiomes is expressed in measurable changes in greenhouse gas production, redox persistence, and nutrient removal performance.

Under land-use conversion to aquaculture ponds, methane production potential declined from 45.2 to 20.1 ng·g^−1^·d^−1^ [13]. This change coincided with reductions in sediment substrate availability, with soil organic carbon decreasing from ~17.6 to 6.97 g·kg^−1^ and C/N ratios declining from ~10.85 to 5.66 [33]. Methane production potential was negatively predicted by sulfate concentration [9],[13]. Consistent with sulfur pathway redistribution described in Section 3.3, sulfate-enriched sediments showed reduced methane production potential. Although lower methane flux may reduce greenhouse gas emissions, this shift indicates intensified reduced-state dominance and enhanced sulfide generation [6], with implications for sediment toxicity and carbon cycling balance in aquaculture systems.

In enriched fish-farm sediments, seasonal sulfate reduction increased by more than an order of magnitude and was accompanied by millimolar sulfide accumulation and sulfate depletion in surface porewaters [6]. These measurements document intensified anaerobic mineralization and reduced oxidative buffering.

Carbon mineralization sensitivity to warming has been quantified in pond sediments across incubation gradients. CO_2_ temperature sensitivity increased (activation energy 0.183 ± 0.036 eV; χ^2^ = 8.7, P = 0.003), whereas CH₄ did not show significant temperature sensitivity (χ^2^ = 3.3, P = 0.06) [12]. These differential responses indicate pathway-specific thermal sensitivity.

Additional sediment investigations report statistically supported differences in gas production and nutrient turnover under aquaculture-relevant disturbance regimes [23],[58],[59].

Nitrogen removal performance is likewise quantifiable. Sediment packed-bed systems achieved 88–90% nitrogen removal under controlled loading [60]. In pond time series, enrichment-linked oxygen decline coincided with altered inorganic nitrogen pools [7], reflecting measurable shifts in nitrogen processing performance under reduced oxygen availability.

Together, these observations demonstrate altered CH_4_ production potential, intensified sulfate reduction and sulfide accumulation, thermally sensitive CO_2_ emissions, and variable nitrogen removal efficiency in aquaculture sediments exposed to climate-relevant forcing.

### 4.2. Increased disease risk for cultured species

Thermal elevation and oxygen limitation translate into measurable increases in pathogen performance and host physiological stress.

Ecosystem-scale datasets report temperature-associated increases in pathogenic *Vibrio* species, including *V*. *vulnificus*, *V*. *parahaemolyticus*, and *V. alginolyticus*, whose abundance and geographic distribution correlate with long-term warming trends [15]. In aquaculture ponds, dissolved oxygen frequently approaches species-specific physiological limits. Critical oxygen partial pressure (Pcrit) in Nile tilapia (*Oreochromis niloticua*) has been reported at approximately 7.7 kPa [61], while pond exposures include sustained hypoxia near 8 kPa and diel minima approaching 0 kPa [25].

Temperature-dependent virulence has been quantified for *Streptococcus agalactiae* infection in Nile tilapia. *In vitro*, log-phase growth was reached faster at 35 °C than at 28 °C (4 h vs 7 h), and bacterial survival in tilapia whole blood differed markedly (97% at 35 °C vs 2% at 28 °C). Hemolytic activity was approximately fivefold higher at 35 °C. Virulence-associated genes (*cylE*, *cfb*, *PI-2b*) showed stronger expression at elevated temperature. *In vivo* challenge experiments resulted in accumulated mortalities of ~85% at 35 °C *vs*. ~45% at 28 °C, accompanied by 30–40-fold upregulation of *cox-2*, *IL-1β*, and *TNF-α* between 6 and 96 h post-infection [62]. Temperature-dependent increases in virulence have also been demonstrated for other aquaculture pathogens, including *Flavobacterium columnare*, where elevated temperature enhanced bacterial growth and disease severity in fish [63].

Accumulation of reduced metabolites such as sulfide and organic acids under enrichment conditions [6] imposes physiological stress on cultured organisms. Elevated sulfide concentrations further constrain aerobic respiratory efficiency under hypoxic conditions, exacerbating metabolic limitation in fish exposed to oxygen decline [4],[61]. Sublethal exposure to combined hypoxia and reduced-state metabolites may suppress feeding performance and increase vulnerability to opportunistic pathogens [62]. Under warming-induced hypoxia, these physiological constraints may compound pathogen-related mortality. Pond time series further document enrichment-linked shifts in inorganic nitrogen pools [7], contributing to eutrophication dynamics that intensify oxygen stress and disease susceptibility [64]–[66].

Environmental ARG enrichment expands resistance reservoirs in aquaculture sediments, representing an additional risk dimension. Comparative resistome analyses demonstrate anthropogenic reshaping of ARG structure and transmission potential [67],[68]. Elevated ARG abundance increases the probability that opportunistic pathogens harbor resistance determinants, complicating outbreak management.

Under combined warming, oxygen limitation, and enrichment, microbial restructuring is therefore associated with higher pathogen proliferation, amplified host inflammatory response, and expanded antimicrobial resistance reservoirs.

### 4.3. Impacts on system productivity and stability

Microbial and biogeochemical shifts translate into measurable production constraints.

In aquaculture systems, hypoxia frequently emerges under conditions of high organic loading and reduced oxygen solubility [3],[4],[66]. Contraction of oxic penetration depth promotes sulfate reduction and fermentative pathways [9],[18], intensifying sulfide accumulation and altering nitrogen routing. These feedbacks increase the probability of recurrent oxygen stress across production cycles and reduce system stability margins.

In Nile tilapia exposed to sustained hypoxia, feed conversion ratio increased from 1.33 under normoxia to 1.44 under hypoxic conditions [25], indicating reduced feed efficiency. Elevated temperature amplifies pathogen virulence and mortality risk [62], increasing production losses during heat events.

Oxygen limitation lowers sediment buffering capacity, increasing the likelihood of hypoxic episodes. Internal nitrogen retention can amplify nutrient loading within ponds, reinforcing oxygen depletion during bloom events [7],[64],[65].

Systems operating under high organic loading and constrained hydrodynamics exhibit greater sensitivity to disturbance [58],[69]. Enrichment-linked restructuring reduces predictability of waste-processing performance under additional perturbations such as feed pulses or thermal extremes [42].

Collectively, these pressures reduce stability margins by increasing vulnerability to oxygen stress, disease outbreaks, feed inefficiency, and treatment failure under climate-relevant forcing. Although evidence density remains highest for marine and coastal sediments, analogous patterns of redox compression, nitrogen redistribution, and productivity constraint are increasingly documented in brackish and freshwater systems. Cross-system differences in microbial responses and measured outcomes are summarized in Table 1.

**Table 1.** Comparative microbial restructuring and measured functional outcomes across marine, brackish, and freshwater aquaculture sediment systems exposed to climate-relevant stressors. The table highlights dominant environmental drivers, observed community-level responses, and quantified ecosystem or production consequences reported in representative studies. Evidence density remains highest for marine and coastal sediments, whereas high-resolution microbial datasets from freshwater pond systems are comparatively limited.

System type	Dominant climate/Production stressors	Observed microbial restructuring	Measured functional outcomes	References
Marine & coastal sediments (cage systems)	Warming, organic deposition, bottom-water hypoxia	Increased sulfate-reducing bacteria (SRB); enhanced *dsrAB* abundance; deterministic clustering under warming; reduced network connectivity	Millimolar sulfide accumulation; increased sulfate reduction rates; altered CO_2_ temperature sensitivity	[6],[12],[21],[22],[32],[42]
Brackish pond systems	Salinity fluctuation, nutrient loading, seasonal hypoxia	Methanogen restructuring; sulfate–methane competitive redistribution; nitrogen pathway shifts	Methane production decline (45.2 → 20.1 ng·g^−1^·d^−1^); altered sediment C/N ratios	[13],[33]
Freshwater pond aquaculture	High organic feed input, diel oxygen oscillation, warming	Decline in nitrifier-associated taxa; enrichment-linked compositional shifts	Increased feed conversion ratio under hypoxia (1.33 → 1.44); altered inorganic nitrogen pools	[7],[25]

## 5. Microbial indicators of climate impact

### 5.1. Taxonomic indicators of environmental stress

Taxonomic restructuring provides reproducible structural signals of environmental forcing across sediment systems. Large-scale sequence surveys demonstrate statistically resolvable community partitioning along salinity, oxygen, and enrichment gradients [5],[27],[29],[30],[42]. Declines in α-diversity and strengthening deterministic clustering under warming (F₉,₅₆ = 27.06, P < 0.001; βNTI > +2) provide quantifiable structural thresholds [12],[22].

Consistent with the redox compression dynamics described earlier, sulfate-reducing bacteria (SRB), particularly Desulfobacterota, function as reliable indicators of reduced-state dominance [21],[70],[71]. Because their expansion tracks reduced-state dominance, proportional SRB abundance can function as a redox indicator. Ratio-based metrics such as SRB:SOB (sulfur-oxidizing bacteria) provide improved sensitivity relative to absolute abundance alone.

Nitrifier-associated taxa decline predictably under oxygen limitation and along freshwater-to-marine transitions [5],[27]. Ratios such as nitrifier:denitrifier or nitrifier:total bacterial abundance may therefore serve as early-warning indicators of declining oxidative capacity. When paired with *amoA* quantification, detection sensitivity increases.

Methanogenic Archaea exhibit statistically significant co-occurrence simplification under salinity conversion [13]. Moreover, where network inference is available, connectivity or modularity metrics can provide supplementary structural indicators of stress.

Temperature-sensitive opportunistic genus such as *Vibrio* show warming-associated expansion [15]. Because pathogen enrichment may precede clinical detection, monitoring *Vibrio* abundance offers disease-relevant early warning under thermal stress.

Across aquaculture systems, enrichment regimes produce reproducible shifts toward anaerobic and fermentative guild dominance with concurrent reductions in network complexity [42],[43]. Composite indices comparing aerobic *vs*. obligate anaerobic guild proportions provide integrated measures of redox compression.

Operational feasibility depends on detection platform. Amplicon sequencing enables broad structural profiling but provides relative abundance estimates that may limit quantitative threshold interpretation [8]. Targeted assays directed at SRB-specific *16S* regions, *Vibrio* markers, or functional genes such as *amoA* enable absolute quantification and improved cross-system comparability [16],[72].

Because single-taxon indicators may lack specificity under compound stress, composite or ratio-based metrics (e.g., SRB:SOB, nitrifier:denitrifier, and aerobic:anaerobic guild proportion) provide greater robustness across environmental contexts.

### 5.2. Functional gene markers as biomarkers

Functional gene abundance provides process-aligned biomarkers that complement taxonomic structure.

Sulfur-cycling genes respond consistently to redox shifts [10]. Quantification of *dsrAB* and *sox* genes enables calculation of *dsrAB*:*sox* ratios as an index of reductive versus oxidative sulfur cycling capacity. Absolute *dsrAB* copy number per gram sediment, or normalized to *16S rRNA* gene abundance, can serve as a proxy of reduced-state intensity.

Nitrogen transformation genes provide complementary resolution. Ratios such as *nirK*/*nirS*:*nrfA* index nitrogen removal versus retention potential [11],[18]. The *nosZ*:*nir* ratio provides an indicator of denitrification completeness and potential N_2_O accumulation risk. Because nitrogen cycling genes respond rapidly to oxygen fluctuations and loading changes [7], gene-level shifts may precede detectable changes in bulk nitrogen pools.

Furthermore, carbon mineralization sensitivity to warming is reflected in altered representation of carbon-degradation pathways [44]–[48]. While no single thermal marker exists, composite indices integrating carbon-degradation and respiration-related genes can provide functional signatures of metabolic redistribution.

ARGs function as cumulative stress biomarkers. Enrichment of multiple ARG classes under nutrient–metal coupling [14] and aquaculture-associated regimes [16],[54],[55],[57],[67],[68] demonstrates responsiveness to anthropogenic pressure. Moreover, normalization of ARG abundance to total bacterial load (ARG:*16S* ratio) provides a scalable index of resistance reservoir intensity independent of biomass variation.

Multi-omics profiling provides additional resolution. Quantitative metaproteomic allocation (823 protein groups; 6,179 peptides; ~9% energy production and conversion) [50], metabolite coverage benchmarks (146 metabolites) [52], and isotopic nitrogen tracing [51] can validate gene-level interpretations at expression and metabolic levels.

For deployment, feasibility and standardization are critical. Shotgun metagenomics enables pathway-level resolution but requires substantial sequencing depth and bioinformatic capacity [8],[16]. Targeted real-time PCR (qPCR) enables cost-effective absolute quantification of *dsrAB*, *nirS*/*nirK*, *nosZ*, *nrfA*, *amoA*, and integron-associated ARGs [14],[72]. Droplet digital PCR (ddPCR) improves precision for low-abundance targets and reduces dependence on calibration curves through partition-based absolute quantification [73]. Consistent normalization per gram sediment and per *16S rRNA* gene copy number is required for cross-system comparability [14],[16].

Composite biomarker panels integrating sulfur, nitrogen, carbon, and resistance metrics, interpreted through ratio-based indices, provide greater diagnostic robustness under interacting climate-relevant pressures than single-gene approaches.

### 5.3. Toward an integrated monitoring framework

No single indicator captures the full spectrum of climate-driven sediment restructuring, andeffective monitoring requires integration across structural, functional, and process-aligned metrics.

A three-tier architecture could be operationalized:

Tier 1 (Structural surveillance): Taxonomic indicators capture compositional deviation from baseline, including α-diversity compression, deterministic clustering thresholds (βNTI > +2) [12], enrichment of sulfate reducers, depletion of nitrifiers, and expansion of opportunistic taxa [5],[27],[42].

Tier 2 (Functional gene indices): Gene-based ratios (*dsrAB*:*sox*, *nir*:*nrf*, *nosZ*:*nir*, ARG:*16S*) quantify shifts in sulfur balance, nitrogen routing, denitrification completeness, and resistance burden [10],[11],[14].

Tier 3 (Process-aligned validation): Process-level benchmarks such as methane production potential, CO_2_ activation energy, nitrogen removal efficiency, and inorganic nitrogen pool dynamics provide calibration of gene-based thresholds [12],[13],[33],[60].

Predictive utility requires system-specific baselines and defined deviation thresholds. Thresholds should be calibrated per system rather than universally imposed, given variability across freshwater, brackish, and marine environments. Ratio-based indices improve cross-system interpretability by reducing dependence on absolute abundance shifts.

Routine monitoring may employ targeted molecular panels (e.g., qPCR and ddPCR) for selected structural and functional markers [14],[72],[73], supplemented by periodic metagenomic surveys for recalibration and pathway-level resolution [8],[16]. Moreover, standardized reporting units and normalization strategies are essential for cross-system comparability and meta-analytic scaling [14],[16].

Integrated monitoring thus links structural compression, functional routing imbalance, resistance expansion, and process-level validation within a unified early-warning framework capable of detecting instability before overt functional impairment.

### 5.4. Emerging predictive frameworks and early-warning signals of sediment instability

In Section 5.3, we outline operational monitoring architecture, though predictive management requires analytical frameworks capable of detecting early-warning signals preceding functional collapse. In ecological systems, transitions toward alternative stable states are often preceded by early-warning signals (EWS), including rising variance, increasing temporal autocorrelation, reduced recovery rates following disturbance (critical slowing down), and loss of interaction redundancy. Although not systematically implemented in aquaculture sediments, several microbial and biogeochemical metrics discussed above exhibit properties consistent with EWS theory.

Structural indicators derived from microbial interaction networks represent one potential signal domain. Temporal reductions in modularity, connectivity, or interaction redundancy may reflect declining functional buffering capacity within sediment microbiomes. When such structural simplification coincides with increasing environmental filtering, these trends may indicate a transition toward reduced-state microbial consortia prior to overt hypoxia or sulfide accumulation.

Functional gene indicators provide a complementary early-warning layer linking microbial structure with ecosystem processes. Divergence or increasing temporal variance in ratios among genes associated with alternative redox pathways, such as *dsrAB* relative to sulfur oxidation markers or *nir*:*nrf* ratios within nitrogen cycling pathways, may indicate progressive redox compression before bulk nutrient pools shift substantially. Similarly, sustained increases in ARG:*16S* ratios may the signal expansion of environmental resistance reservoirs under cumulative environmental pressures. Temporal trajectories of these indicators are typically more informative than single measurements.

Process-level indicators may also display early-warning behavior. Increasing recurrence of oxygen minima under warming or altered temperature sensitivity of sediment carbon mineralization may precede persistent hypoxic regimes. Rising temporal autocorrelation in dissolved oxygen or sediment redox potential may indicate slowing recovery rates following disturbance, which is consistent with critical slowing.

Advances in metagenomic monitoring provide practical approaches for operationalizing such early-warning indicators in aquaculture systems. High-throughput sequencing enables detailed characterization of microbial community composition and metabolic potential, enabling microbial indicators to be linked with water-quality dynamics and pathogen emergence. Metagenomic surveys of aquaculture sediments illustrate the scale at which such monitoring can operate, while genome-resolved metagenomic analyses demonstrate how microbial community structure can be linked quantitatively to ecosystem function. In addition to nutrient-cycling processes, environmental microbiomes act as reservoirs of antibiotic resistance genes whose abundance may increase under interacting stressors such as warming, pollutants, and microplastic-associated biofilms that enhance horizontal gene transfer. Integrating microbial genomic indicators with environmental monitoring frameworks may therefore enable earlier detection of ecological destabilization than conventional physicochemical measurements alone.

In practice, monitoring frameworks may integrate several complementary classes of indicators. These include community-structure indicators (taxonomic composition and diversity indices), functional gene indicators (e.g., *dsrAB*, *nirS*/*nirK*, and *amoA* abundance), network-structure indicators (changes in connectivity or modularity), and process-rate indicators derived from geochemical measurements or isotope-based assays. Tracking temporal trajectories of these indicators alongside environmental variables such as oxygen concentration, temperature, and organic loading may reveal microbial transitions preceding hypoxia, sulfide accumulation, or pathogen proliferation.

Predictive integration requires coupling these indicators with quantitative ecological models. Parameterization of oxygen kinetics, organic loading, and microbial assembly thresholds enables simulation of nonlinear transitions under interacting stressors. Statistical learning approaches applied to high-dimensional microbial datasets may assist in identifying indicator combinations that precede hypoxic episodes or productivity decline. However, predictive reliability depends on longitudinal sampling, system-specific baseline calibration, and standardized normalization strategies.

Multi-omics integration further enhances early detection capacity. Shifts in metaproteomic allocation, metabolite profiles, or isotopic nitrogen redistribution may capture metabolic reorganization prior to detectable compositional turnover. Embedding these layers within time-series monitoring frameworks strengthens detection of functional transitions.

Collectively, integration of microbial structural indicators, functional gene trajectories, process-level metrics, and predictive modeling provides a structured pathway toward anticipatory management of aquaculture sediments under interacting environmental stressors.

## 6. Toward climate-resilient aquaculture

### 6.1. Integrated multi-trophic aquaculture (IMTA) as a stabilizing strategy

Integrated multi-trophic aquaculture (IMTA) functions as a nutrient-buffering architecture by redistributing dissolved and particulate waste streams across trophic levels [74]–[76].

Seaweeds assimilate dissolved inorganic nitrogen and phosphorus, reducing accumulation in the water column. Suspension-feeding bivalves intercept particulate organic matter prior to sediment deposition. Deposit feeders enhance sediment mixing and oxygen penetration through bioturbation. These combined processes reduce sediment organic loading intensity and moderate oxygen demand [6],[20],[77].

Quantitative analyses indicate that extractive components can remove substantial fractions of dissolved inorganic nitrogen in salmon–seaweed systems [74]. Life-cycle modeling demonstrates measurable reductions in nutrient discharge intensity and carbon footprint under integrated designs [58],[78].

Operational performance depends on hydrodynamic context, biomass balancing, and trophic compatibility. IMTA is most effective in coastal systems with sufficient water exchange. In pond-based aquaculture, partial analogues include macrophyte co-culture and filter-feeder integration.

IMTA therefore operates as a flux-redistribution strategy that reduces depositional intensity and stabilizes nutrient dynamics under high production loads.

### 6.2. Harnessing microbiome management

Microbiome management operates directly on microbial functional capacity within production systems.

Probiotic and bioaugmentation strategies introduce microbial taxa with defined metabolic functions, including nitrifiers, denitrifiers, and heterotrophic degraders. Operational dosing typically ranges from 10⁴–10¹⁰ CFU g⁻¹ feed, depending on formulation and delivery route. Experimental studies demonstrate measurable pathogen suppression and host performance responses following probiotic supplementation [79]–[82]. For example, gut-associated *Paenibacillus polymyxa* isolates inhibit major fish pathogens, including *Aeromonas hydrophila* and *Pseudomonas fluorescens* with inhibition zones exceeding 25 mm while producing digestive enzymes such as amylase (~266 U mL⁻¹) and protease (~75 U mL⁻¹) that may support nutrient assimilation [82]. Bacillus- and lactic-acid-bacteria-based probiotic supplementation has been associated with improved immune parameters, enhanced growth performance, and increased disease resistance in shrimp and finfish aquaculture systems [79],[81]. Additional feeding trials and microbiome-management studies report improved growth performance, modulation of gut microbial composition, and reduced pathogen dominance in cultured fish and shrimp production systems [34],[83]. For example, a mangrove-derived probiotic *Streptomyces* inhibited *Vibrio parahaemolyticus*, reducing pathogen viability by 28–35% in co-culture assays, while probiotic supplementation increased survival of challenged giant freshwater prawn (*Macrobrachium rosenbergii*) larvae threefold and improved growth performance by ~17% under experimental conditions [80].

High-throughput sequencing enables comprehensive characterization of microbial community structure and metabolic potential in aquaculture environments [84]. Metagenomic surveys of aquaculture sediments demonstrate the scale at which such monitoring can operate; for example, a collection of 30 sediment metagenomes from estuarine aquaculture farms across four estuaries was generated to characterize microbial diversity and resistome composition in shellfish and sea bream production systems [85]. Genome-resolved metagenomic analysis in aquatic environments has reconstructed hundreds of microbial genomes (523 metagenome-assembled genomes across 34 bacterial and archaeal phyla) linked to nitrogen and sulfur cycling processes across oxygen gradients, illustrating how microbial community composition can be quantitatively linked to ecosystem biogeochemical function [86]. Environmental microbiomes also serve as reservoirs for antibiotic resistance genes, whose abundance can increase under interacting stressors such as warming, pollutants, and microplastics that promote biofilm formation and horizontal gene transfer within microbial communities [87].

Biofloc technology represents an endogenous microbiome modulation strategy. Maintaining elevated carbon-to-nitrogen ratios, typically >10–15:1, promotes heterotrophic assimilation of ammonium into microbial biomass. Empirical studies demonstrate improved nitrogen retention efficiency, reduced water exchange requirements, and enhanced production efficiency under controlled biofloc regimes [78],[88],[89].

Sediment drying and fallowing between production cycles oxidize reduced compounds and reduce pathogen persistence prior to restocking [6],[77]. These practices are widely implemented in semi-intensive pond systems and are operationally low-cost relative to continuous aeration.

Intervention performance should be evaluated using measurable metrics such as nitrogen removal efficiency, sulfide reduction, pathogen load, and feed conversion ratio, rather than compositional shifts alone.

Microbiome management therefore provides a scalable strategy to stabilize nutrient cycling and pathogen dynamics under variable environmental conditions.

### 6.3. Engineering solutions

Engineering interventions regulate oxygen supply, substrate flux, and sediment–water exchange under intensive production.

In pond systems, feeding inputs commonly operate at ~1–2% biomass per day, and total suspended solids can reach several hundred mg·L^−1^ under biofloc-type regimes [78]. These magnitudes determine oxygen demand and organic loading intensity.

Mechanical aeration increases dissolved oxygen, enhances vertical mixing, and reduces stratification. Oxygenation strategies act mechanistically by expanding oxic penetration depth and reducing conditions that favor sulfate-reducing metabolism, thereby restoring aerobic nitrogen transformation pathways rather than solely elevating bulk dissolved oxygen concentrations [6],[18]. By improving oxidative buffering capacity, aeration disrupts the feedback loop between organic loading, sulfide accumulation, and recurrent hypoxia [4]. Paddlewheel aerators and diffused-air systems maintain oxygen above species-specific physiological thresholds. For example, in Nile tilapia, controlled pond experiments demonstrated that sustained aeration improved feed conversion ratios and reduced mortality compared with hypoxic conditions [25].

Hydrodynamic optimization reduces localized organic accumulation. In coastal cage systems, site selection based on current velocity and depth minimizes particulate deposition beneath cages. In pond systems, circulation design and directional flow redistribute solids and reduce depositional hotspots. Appropriate hydraulic residence time moderates nutrient accumulation and diel oxygen oscillations [64],[65].

Sediment management practices, including sludge removal and constructed sedimentation basins, intercept particulate wastes prior to burial. Mechanical extraction or drying between cycles reduces labile organic substrate pools [6],[77].

In recirculating aquaculture systems, engineered biofilters such as trickling filters and moving bed biofilm reactors stabilize nitrification capacity under variable loading. Performance benchmarks include nitrogen removal efficiency targets under defined loading rates [60].

Engineering interventions are immediately deployable but energy-intensive. Effectiveness depends on system sizing relative to biomass density, maintenance during thermal extremes, and alignment with feeding intensity.

### 6.4. The critical role of monitoring and adaptive management

Resilience strategies require defined intervention triggers linked to measurable system deviation.

Routine monitoring should include dissolved oxygen profiling (including diel minima), sediment redox potential, inorganic nitrogen concentrations (NH_4_^+^, NO_2_^-^ and NO_3_^-^), and solids accumulation. Periodic microbial indicator panels (Section 5) provide additional resolution of sulfur balance, nitrogen routing, and resistance burden.

Adaptive responses may include aeration intensification, feeding reduction, carbon adjustment in biofloc systems, sludge removal, drying cycles, probiotic supplementation, or biosecurity reinforcement. Early intervention during deviation from baseline is more effective than corrective action after prolonged instability.

Thresholds should be calibrated per system based on historical variability, biomass density, and hydrodynamic context rather than universally imposed.

Integrated implementation links ecological design (IMTA), microbiome steering, engineering controls, and indicator-guided decision rules within a unified management cycle.

Climate resilience therefore depends on coordinated control across nutrient interception, microbial stabilization, infrastructure capacity, and adaptive governance.

## 7. Concluding remarks and future perspectives

The evidence synthesized in this review demonstrates that climate-relevant forcing restructures sediment microbiomes across compositional, interaction, and functional layers. However, several critical uncertainties limit predictive translation.

First, cross-system comparability of effect sizes remains limited. Temperature increments are often precisely quantified, whereas salinity amplitudes (PSU shifts), oxygen minima duration, and nutrient loading intensities are inconsistently standardized across freshwater, brackish, and marine systems. This restricts threshold generalization and limits predictive transferability between production contexts.

Second, compound-stressor dynamics remain insufficiently resolved. Most experimental designs examine paired drivers, yet operational systems experience simultaneous thermal, redox, nutrient, and hydrodynamic forcing. Nonlinear interaction structures, including tipping thresholds, hysteresis behavior, and recovery trajectories under repeated disturbance cycles, remain poorly constrained [22],[24],[28].

Third, temporal persistence and reversibility are not well quantified. While legacy effects and trajectory dependence are evident [22],[40],[41], the time required for functional reversion after stress relaxation is rarely measured. It remains unclear whether repeated seasonal forcing progressively shifts baselines toward alternative stable configurations or whether buffering capacity can be maintained under managed regimes.

Fourth, attribution of resistome expansion under climate forcing requires sharper resolution. Although enrichment and metal coupling are associated with antimicrobial resistance gene proliferation [14],[16],[54],[55], the relative contributions of thermal selection, oxygen compression, horizontal gene transfer frequency, and anthropogenic loading intensity remain difficult to disentangle [53],[57],[67],[68].

A major unresolved dimension is the quantitative role of horizontal gene transfer under interacting warming–hypoxia–enrichment gradients. Genomic-context evidence demonstrates ARG co-localization with integrons and mobile elements [14],[16],[53]–[55], yet transfer frequencies under multifactorial climate-relevant forcing have not been experimentally resolved. Distinguishing clonal expansion from gene-centric dissemination requires plasmid-resolved metagenomics, long-read assembly of mobile genetic elements, integron cassette mapping, and controlled conjugation assays under defined multi-driver gradients within the broader framework of bacterial genomic adaptability [56].

Fifth, predictive modeling capacity lags behind descriptive insight. There is a deficit of coupled ecological–biogeochemical models integrating thermal sensitivity, oxygen kinetics, nutrient routing, microbial assembly dynamics, and resistance reservoir expansion. Moreover, threshold behavior under extreme heat and hypoxia events is insufficiently parameterized for operational risk forecasting.

Addressing these gaps will require multifactorial experimental designs manipulating temperature, oxygen, salinity, and loading simultaneously under controlled gradients, alongside longitudinal field datasets spanning multiple production cycles. Harmonization of environmental units and reporting standards will be essential for meta-analytic scaling and cross-system inference.

Future progress depends on transitioning from descriptive association to quantitative threshold identification and model validation. Without standardized stressor quantification, compound-driver experiments, and mobilome-resolved resistome tracking, predictive management of aquaculture sediment microbiomes under climate change will remain constrained.

## Use of AI tools declaration

The authors state that they have not utilized Artificial Intelligence (AI) tools when creating this article.
